# Face masks and COVID-19: don’t let perfect be the enemy of good

**DOI:** 10.2807/1560-7917.ES.2020.25.49.2001998

**Published:** 2020-12-10

**Authors:** Benjamin J Cowling, Gabriel M Leung

**Affiliations:** 1 WHO Collaborating Centre for Infectious Disease Epidemiology and Control, School of Public Health, LKS Faculty of Medicine, The University of Hong Kong, Hong Kong Special Administrative Region, China; 2 Laboratory of Data Discovery for Health, Hong Kong Science and Technology Park, Hong Kong Special Administrative Region, China

**Keywords:** Covid-19, face mask

Whether to don face masks or facial coverings to prevent community spread of coronavirus disease 2019 (COVID-19) has perhaps been one of the most argued and divisive issues, initially between East Asia and the West and later within western countries. Even the World Health Organization (WHO) had prevaricated on the issue during the initial months of the pandemic until it advised governments to ‘encourage the general public to wear masks in specific situations and settings as part of a comprehensive approach to suppress COVID-19 transmission’ in guidance issued in June 2020 [1].

To assess the appropriateness of masks as an intervention measure, it is important to first understand the aerobiology and modes of transmission of COVID-19. There is general agreement that transmission risk is increased with prolonged close contact. The question concerning longer-range aerosol transmission remains a subject of strong debate. Severe acute respiratory syndrome coronavirus 2 (SARS-CoV-2) RNA detection in aerosols has been reported in some retrospective case reviews [2,3] and viable virus could be detected in laboratory experiments [4] and patient settings [5] as well as anecdotal reports such as a reported restaurant outbreak [6]. While definitive proof of aerosol transmission remains elusive, prominent scientists have advocated a precautionary approach to mitigate the risks of aerosol spread, noting in particular that the risk of aerosol transmission would be greatest at short range [7,8].

Widespread use of face masks may reduce community transmission in two ways. First, through source control, since masks worn by persons who are infected and contagious can effectively reduce viral dissemination into the environment [9,10]. This can be particularly important in the context of pre-symptomatic transmission of COVID-19 [11,12]. Second, face masks can have an impact through protection of uninfected persons, since masks can effectively filter virus-laden particles from the air breathed in [13-15]. However, there are also caveats. Masks will not be worn 100% of the time – they will not generally be worn in households or in some social settings, and they will not be worn while eating. In addition, even when masks are worn, they should reduce the risk of transmission but they may not completely eliminate transmission. While most research on face masks has involved surgical type face masks, it should be presumed that reusable cloth masks could provide similar benefits if they have a sufficient number of layers and preferably a filter.

While there is mechanistic support for the effectiveness of face masks from laboratory-based studies, evidence from real-life studies can confirm whether mask policies could have an impact on community transmission. The highest quality of scientific evidence on the real-life efficacy or effectiveness of an intervention is provided by randomised controlled trials. A number of randomised trials of face masks have been done to prevent transmission of respiratory virus infections. For example, the 2019 WHO guidance on non-pharmaceutical interventions cited evidence from 14 randomised controlled trials that did not support a statistically significant effect on transmission of laboratory-confirmed influenza [16]. However, in that guidance, mechanistic evidence of the effectiveness of face masks was used as a basis for a recommendation for widespread mask use in the community in influenza epidemics/pandemics of high or extraordinarily high severity [16].

In this issue of *Eurosurveillance*, Brainard et al. reviewed 12 randomised trials and 21 observational studies of the effectiveness of face mask use against respiratory virus transmission [17]. The meta-analysis of randomised trials has similar findings to a number of earlier Cochrane reviews [18-21] and published systematic reviews and meta-analyses [22-37], namely that face mask interventions could probably reduce transmission by a small margin but not a large margin in the community. Brainard et al. estimate that masks reduce the risk of infection by around 6% to 15% [17]. While randomised trials typically provide the highest quality of evidence on interventions, limitations of trials in face masks have included the lack of blinding, and adherence with the intervention leading to effect dilution.

It should be noted that widespread use of face masks in an epidemic will have greater community benefit through reducing contagiousness of infected persons in addition to protecting susceptible wearers. One recent study from Germany reports a 45% reduction in transmission through face mask use [38], although this study might have overestimated the impact of masks if other public health measures and behavioural changes occurred simultaneously. There is evidence that universal wearing of face masks has not been sufficient to control COVID-19 transmission and additional public health measures are required. For example, Hong Kong has experienced multiple community epidemics of COVID-19 despite universal face mask use since January 2020 [39]. That said, most large clusters accounting for a substantial proportion of the total case burden have occurred in places where masks are not worn, such as bars, restaurants, gyms, elderly homes and workers’ dormitories [40], while within-household transmission is also a major contributor to overall case numbers.

While most face mask trials have aimed to prevent influenza virus or any respiratory virus transmission, the Danish Study to Assess Face Masks for the Protection Against COVID-19 Infection (DANMASK-19) trial has just reported on the efficacy of masks to prevent COVID-19 transmission [41]. In this trial, 6,024 adults were randomly assigned to a mask recommendation or a control group, and after 1 month the cumulative incidence of COVID-19 in the two groups was 1.8% and 2.1% respectively, with a point estimate of a 15% reduction in risk associated with the face mask recommendation. However, this small risk reduction was not statistically significant. Of note, the study had only been powered to identify a 50% or greater risk reduction. The results of this trial should thus not be interpreted as evidence that masks do not work, since the effect size reported is very consistent with the effects that would be expected based on previous meta-analyses including this new report by Brainard et al. [17] One concern about the trial by Bundgaard et al. is the use of serology to identify outcomes. Since participants were only followed up for one month [17], it is possible that some infections identified in serology at day 30 were actually infections that occurred before the intervention, leading to effect dilution.

In comparison to randomised trials, observational studies provide relatively less reliable information on the effects of interventions, particularly for an intervention such as face masks that are often combined with other protective measures or changes in behaviours. A recent meta-analysis of observational studies found that face mask use by those exposed to infected individuals in non-healthcare observational settings was associated with a 44% risk reduction of infection with severe acute respiratory syndrome coronavirus (SARS-CoV) in 2003 [31]. However, one of the three original studies that formed the summary statistic actually referred to mask use by visiting family members to hospitalised SARS-CoV patients in 2003, thus the exposure per se was healthcare related. In the same review, eye protection alone was estimated to provide 78% reduction in risk of SARS-CoV or Middle East Respiratory Syndrome coronavirus (MERS-CoV) infection [31], an effect size which appears highly implausible given that eyes are unlikely to be a major route of infection.

There are clear evidential gaps in the science of modes of COVID-19 transmission. Nevertheless, there is compelling evidence that masks can contribute to the control of COVID-19. Given that face masks are inexpensive in comparison to the other public health measures being used to control COVID-19, even a limited effect on transmission would justify their widespread use. In addition to recommending that people wear face masks in poorly ventilated, crowded settings or when community prevalence is high, some health authorities might even consider to recommend the practice in all settings when in company. The only caveat relates to potential diversion of scarce supplies for healthcare settings, in which case alternative forms of facial coverings that are made of appropriate materials should be considered [1].
